# Neutrons for Cultural Heritage—Techniques, Sensors, and Detection

**DOI:** 10.3390/s20020502

**Published:** 2020-01-16

**Authors:** Giulia Festa, Giovanni Romanelli, Roberto Senesi, Laura Arcidiacono, Claudia Scatigno, Stewart F. Parker, M. P. M. Marques, Carla Andreani

**Affiliations:** 1 CENTRO FERMI—Museo Storico della Fisica e Centro Studi e Ricerche “Enrico Fermi”, Piazza del Viminale 1, 00184 Rome, Italy; giulia.festa@centrofermi.it (G.F.); roberto.senesi@uniroma2.it (R.S.); claudia.scatigno@uniroma2.it (C.S.); carla.andreani@uniroma2.it (C.A.); 2 ISIS Facility, STFC Rutherford Appleton Laboratory, Chilton, Didcot, Oxfordshire OX11 0QX, UK; stewart.parker@stfc.ac.uk; 3 NAST Centre and Physics Department, Università degli Studi di Roma “Tor Vergata”, Via della Ricerca, Scientifica 1, 00133, Rome, Italy; 4 CNR-IPCF Sezione di Messina, Viale Ferdinando Stagno d’Alcontres 37, 98158 Messina, Italy; 5 Diamond Light Source, Rutherford Appleton Laboratory, Chilton, Didcot, Oxfordshire OX11 0DE, UK; laura.arcidiacono@diamond.ac.uk; 6 UCL, University College of London Institute of Archaeology, 31-34 Gordon Square, Kings Cross, London WC1H 0PY, UK; 7 Química-Física Molecular, University of Coimbra, 3004-535 Coimbra, Portugal; pmc@ci.uc.pt; 8 Department of Life Sciences, University of Coimbra, 3000-456 Coimbra, Portugal

**Keywords:** neutron techniques, cultural heritage, non-destructive and non-invasive characterization

## Abstract

Advances in research in Cultural Heritage see increasing application of a multidisciplinary approach and the combined use of physical and chemical characterization of artefacts that can be used to define their structure and their state of conservation, also providing valuable information in selecting the most suitable microclimatic conditions for the exhibition environment. This approach provides a platform for a synergic collaboration amongst researchers, restorers, conservators, and archaeologists. Existing state-of-the-art technologies for neutron-based methods are currently being applied to the study of objects of historical and cultural interest in several neutron-beam facilities around the world. Such techniques are non-invasive and non-destructive and are, therefore, ideal to provide structural information about artefacts, such as their composition, presence of alterations due to the environmental conditions, inclusions, structure of the bulk, manufacturing techniques, and elemental composition, which provide an overall fingerprint of the object’s characteristics, thanks to the nature of the interaction of neutrons with matter. Here, we present an overview of the main neutron methods for the characterization of materials of interest in Cultural Heritage and we provide a brief introduction to the sensors and detectors that are used in this framework. We conclude with some case studies underlining the impact of these applications in different archaeological and historical contexts.

## 1. Introduction

Cultural-Heritage artifacts, in particular ancient ones, still pose many interesting and important challenges, such as the correct determination of their historical and cultural timeframe, their location and method of production, and the choice of suitable treatments and environmental conditions for their restoration and conservation [1,2,3,4]. Amongst the large variety of physical and chemical techniques currently employed for the characterization of ancient objects, neutron-based methods are able to provide unique information, thanks to their particular interaction mechanisms with matter [5,6,7,8]. Neutron beams are used for the quantitative investigation of Cultural Heritage objects of different materials, such as metals, pottery, stone, paintings, and wood [1]. Having no electric charge, neutrons are able to penetrate metal and stone for several centimeters without substantial attenuation, thus providing information regarding the interior of the samples in a non-invasive and non-destructive manner. On the other hand, they cannot be directly detected, which requires neutron sensors that are based on the capture of the neutron by suitable materials, and the detection of the product radiation, e.g., photons or ions. Neutrons also possess a high sensitivity to light nuclei, particularly hydrogen, and they can help in distinguishing between isotopes of the same element and also neighboring elements in the periodic table. Caveats exist, of course. When using neutrons, some isotopes in the samples become active (particularly so for metals), and therefore the experimental teams must be careful about handling procedures and irradiation times. The decay times can range from ps to ns, in the case of excited nuclear states of an isotope, or from ms to hundreds of years, for the ground state of unstable isotopes. In this framework, a non-radioactive object, once irradiated, could become radioactive for days (Cu, Au, or Ag), months, or years (e.g., Co). Moreover, one should notice that the safety issues that are related to the use of radiation-based techniques require compliance with national regulations.

In this paper, we briefly review the basic ideas behind the interaction of neutrons with matter, without going into the theoretical details and formalism, which are discussed in detail elsewhere [9], yet having in mind the users of these techniques from the broader scientific community. We also provide an introduction to the sensors and detectors that are routinely used in the application of neutron-based techniques. We then illustrate the uses of neutrons in Cultural Heritage with some recent examples. The reader can find additional discussion and funding opportunities about the application of nuclear-based techniques to the conservation, restoration, provenance, dating, and authenticity verification of Cultural Heritage samples in a recent report of the International Atomic Energy Agency [10].

## 2. Neutron Methods—General Description

**Figure 1:**
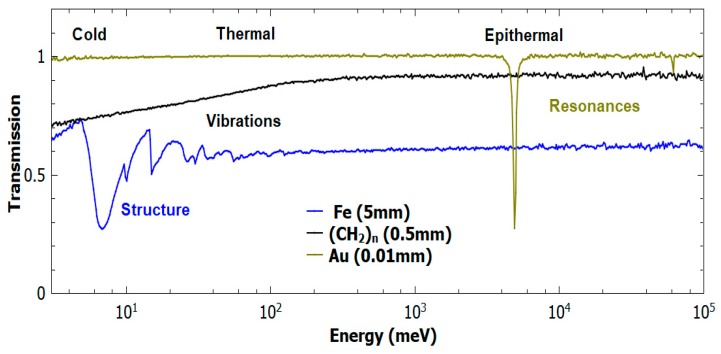
The total neutron cross section of a series of materials as function of the incident neutron energy, as measured on the VESUVIO spectrometer [16]: a 5-mm-thick iron slab (blue), a 0.5-mm-thick polyethylene foil (black) [17] and a 0.01-mm-thick gold foil (gold). The energy ranges for cold, thermal, and epithermal neutrons are also indicated.

Neutron-based techniques started to develop for the non-invasive and non-destructive analysis of bulk materials after the discovery of the neutron by Chadwick in 1932 [11] and the development of methods and technology in neutron production [12]. Neutrons provide a complementary probe to photon-based methods, with the possibility of studying both the structure and dynamics of condensed-matter systems. Over the decades, neutron facilities have experienced continuous development, with new instrumentation and new applications. While the number of electrons of the elements under investigation strictly drives the probability of electromagnetic interaction of X-rays with condensed matter, the neutron interaction is a nuclear process, and the magnitude of the related cross section has no trivial dependence on atomic number [13]. Refs. [14,15] pictorially present the comparison of X-ray and neutron cross sections and attenuation coefficients. In fact, the cross section is both element and isotope dependent. Figure 1 shows some examples of neutron-transmission spectra for several materials, where lower values of the transmission function are related to higher values of the total cross section, i.e., the probability for a neutron to interact with a material. The cross section can be roughly divided into a scattering and a capture contribution.

The structure and dynamics of a system can be studied with neutron scattering. In particular, when a neutron does not change energy during the interaction, the structure of the system is probed in the so-called elastic scattering. Alternatively, the neutron can gain or lose energy as a consequence of the interaction with moving atoms and molecules, and the dynamics of a system can be studied in the so-called inelastic scattering. Moreover, each element is associated with coherent and incoherent parts to the cross section, with purely coherent scatterers that are particularly suitable for elastic scattering. Finally, special attention should be paid to the large incoherent cross section of hydrogen, which is approximately tenfold larger than that of any other common element or isotope, which makes it particularly suitable for inelastic scattering investigations [18].

In addition to the scattering processes, some elements (and isotopes) have particularly large probabilities to absorb the incoming neutrons. One speaks of radiative capture when such absorption is followed by the emission of a gamma-ray cascade. Radiative capture is generally divided into two regions for slow neutrons (neutrons with energies below the MeV region): an absorption region, which is of particular importance for thermal-to-cold neutrons, where the cross section is proportional to the inverse of the neutron’s velocity; and, a resonant region, where the cross section has peaks at well-defined neutron energies, especially in the epithermal region (Figure 1). The energy values for such resonances are fingerprints of different elements and isotopes [19].

For a neutron to be detected, it needs to be captured or absorbed, rather than just scattered. Coherent elastic scattering is often used, before the neutron detection, in order to select the energy of neutrons in a given region of interest [9,20], while incoherent inelastic scattering can be used to lower the neutron energy and increase the probability for the moderated neutrons to be absorbed. On the other hand, the capture of neutrons is needed to allow for their detection. Thus, neutron sensors are rich in elements (and in particular isotopes) with large absorption cross sections, such as helium (^3^He), boron (^10^B), lithium (^6^Li), cadmium, or gadolinium [13]. In the case of helium, boron and lithium, the capture process is followed by the emission of charged ions and sensors are coupled with gas electron multipliers [21], or scintillating materials. In the case gadolinium- or cadmium-based sensors, one detects the prompt emission of gamma rays and one can use gamma-sensitive scintillators, high-purity germanium detectors, or even CCDs [22]. We also note that epithermal neutrons can be detected while using a resonant nuclear reaction and measuring the resulting emission of photons [23].

Based on the abovementioned discussion, neutron techniques that are applied to Cultural Heritage can be divided into three main classes: neutron imaging, neutron scattering, and neutron capture techniques.

### 2.1. Neutron Imaging

**Figure 2:**
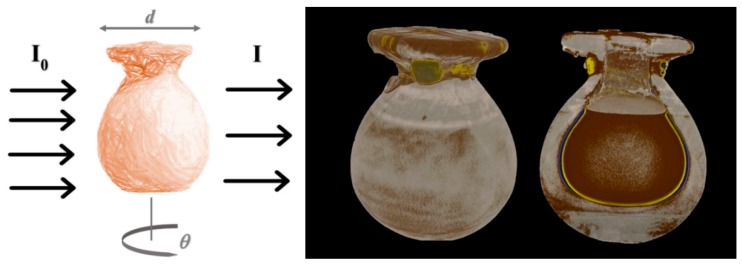
Neutron radiography and tomography. (**Left**) diagram of a standard radiographic and tomography set-up; (**Right**) radiography and tomography on a sealed Egyptian vase [32].

Neutron imaging is based on the use of neutron beams to obtain a radiograph or tomograph of the investigated object, while taking advantage of the particular interaction probability (cross section) with the investigated sample. Neutron radiography is one of the neutron imaging techniques and it is based on the following principle: when a neutron beam passes through an object, the material, as a function of the total cross section, attenuates part of the radiation. The intensity *I*(*x*,*y*) of the transmitted beam is given by:

*I*(*x*,*y*) = *I*_0_(*x*,*y*)*e*^−∫^*path^μ^*^(*x*,*y*,*z*)d*z*^ (1)

where *I*_0_(*x*,*y*) is the incident beam in a plane (*x*,*y*) transversal to the propagation direction *z* and the linear attenuation coefficient, *μ*(*x*,*y*,*z*), is proportional to the depth and density of the object along the *z* direction via the neutron total cross section. In general, *μ* is a function of the incident neutron energy, as is the cross section. However, most of the time the information provided by an imaging camera is summed over all of the neutron energies in the incoming beam [22]. In the case of thermal-neutron beams (tens to hundreds of meV), neutron imaging is particularly suitable for the detection of hydrogenous materials, as shown in Figure 2.

Enhanced information can also be obtained by measuring the energy of the incident neutron, for each pixel in the imaging camera. In this case, referred to as energy-dispersive neutron imaging, one exploits the specific features in the transmission functions of Figure 1. For example, the edges in the low-energy part of the spectrum of iron correspond to the position of its Bragg peaks, as also discussed in the next Section, and are characteristic of the phase and texture of the crystalline domains in a given material. Therefore, by selecting specific regions in the energy-dispersive spectra, one can obtain additional information regarding the structural composition of the sample [24,25], a technique that is also referred to as Bragg–Edge Neutron Imaging. Some examples of the application of this technique are provided in Refs. [26,27,28,29]. In these cases, one takes advantage of the coherent and elastic interaction of neutrons with the sample. A recent example of energy-dispersive neutron imaging that was based on the incoherent and inelastic contribution was reported in [30].

Neutron tomography provides a three-dimensional (3D) reconstruction of the internal features of an object through the acquisition of a large number of radiographs at different angles and the application of reconstruction algorithms [31]. This technique is successfully applied in Cultural Heritage to reconstruct the internal features of a non-homogeneous object and, in particular, for object classes, such as metals, large sealed ceramic vases, or stones, which cannot be investigated by other techniques (such as X-ray-based ones due to the low penetration power in such materials).

### 2.2. Neutron Elastic Scattering

Neutron diffraction techniques are useful tools for studying the structure of materials, spanning the mesoscopic- to the nano-scale. Ancient artefacts are generally made of polycrystalline materials, such as metals, pottery, stones, etc. Neutron diffraction techniques are based on the elastic scattering, where the incident neutron energy is the same as the energy of the scattered neutron beam, and they are used for analyzing the structure of the investigated material. Bragg’s law describes the diffraction process:

*nλ* = 2*d_hkl_*sin*θ* (2)

where *λ* is the wavelength of the incident neutron beam, *n* is a positive integer, *d_hkl_* is the distance between lattice planes in a crystalline structure, and *θ* is the angle between the crystalline plane and the incident neutron beam. Neutron diffraction can be carried out at both reactors and spallation neutron sources [33].

**Figure 3:**
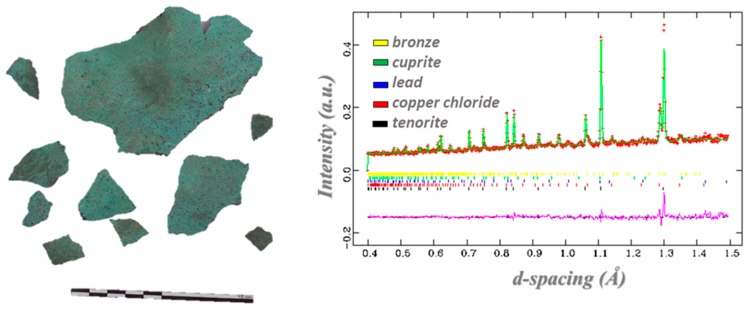
Neutron diffraction results (**Right**) on an Etruscan metallic bronze fragment (**Left**) [51].

The peak positions in the diffraction pattern are directly related to the crystal lattice geometry and dimensions, while the peak intensities are determined by the atomic arrangement in the unit cell and they are related to the structure factor by the following formula:

*I_hkl_* ∝ |*F*_*hk*l_|^2^ = |∑*_j_ b_j_*⋅e^2π*i*(*h⋅x*^*_j_*^+*k⋅y*^*_j_*^+*l⋅z*^*_j_*)|^2^ (3)

where (*h*,*k*,*l*) are the Miller indexes of the lattice planes, *b_j_* is the neutron scattering length of atom *j* in the sample, and (*x*,*y*,*z*) define the atomic position in the unit cell. Structural information at different scales is extracted through the analysis of diffraction patterns, such as phase structure, crystal structure that is related to the atomic arrangement of each phase, grain structure (named texture) providing the size, shape, and orientation of grains and micro-structures. Of particular interest in this framework is the analysis of residual stress and strain in samples subject to mechanical or thermal treatments [5,34,35]. Quantitative phase analysis is carried out through the identification of the phases in the sample by comparison of the experimental diffraction patterns with the diffraction pattern of known compounds, as reported in databases, such as PDF [36], ICSD [37], and American Mineralogist [38]. After the phase identification, experimental diffraction patterns are refined through the Rietveld method enabling quantitative multi-phase analysis [39,40] through a least-square fitting of the experimental pattern. This provides the phase fractions, structural parameters, micro-structural characterization, and preferred orientation from the analysis of the peak position, peak broadening, and intensity. Figure 3 shows an example of this process, as applied to an Etruscan bronze fragment. Diffraction analysis is performed with dedicated software, such as GSAS [41], MAUD [42], and FULLPROF [43].

Archaeologists and bioanthropologists routinely carry out examination of human remains to retrieve varied information, such as dating or post-mortem changes [44,45,46,47]. In fact, the skeleton is often the only preserved human remain that is found in archaeological settings, having frequently been exposed to heat (e.g., fires, cremations), which often renders DNA identification impossible, due to DNA destruction at high temperatures. Hence, the investigation of these samples can provide valuable evidence regarding the circumstances of death, the environmental setting where the specimens were found, or the habits and funerary practices of ancient civilizations. Neutron diffraction methods have recently been used for the analysis of human skeletal remains, namely bones that are subjected to burning (known to undergo heat-prompted variations [48,49]), with a view of determining the phases and chemical composition of the bone at particular temperatures [50].

Finally, elastic scattering of neutrons at small and ultra-small angles, SANS and USANS, respectively, is used as a non-destructive technique to study the structure of materials at the mesoscopic scale [52]. The study of nanostructured surfactant-based systems for the removal of polymers from wall paintings [53] and the characterization of the porosity of Italian marbles [54] are examples of SANS investigations.

### 2.3. Neutron Spectroscopy

Inelastic neutron scattering (INS) is a vibrational spectroscopy technique, whereby a neutron loses energy by interacting with molecules or lattices. INS is particularly suitable for the analysis of vibrational modes involving hydrogen, owing to its large scattering cross section. Different chemical bonds correspond to specific vibrational frequencies and fingerprint features in the spectra that provide information regarding the structure and dynamics of the system under investigation [55], which is complementary to the data that were delivered by optical vibrational spectroscopy (Fourier transform infrared (FTIR) and Raman). INS methods have been shown to be an invaluable tool for probing bones (both faunal and animal), since they allow for unique access to the hydrogen atoms in the inorganic matrix of this highly heterogeneous material—comprising proteins (mostly collagen I) and lipids woven into an inorganic matrix of hydroxyapatite, Ca_10_(PO_4_)_6_(OH)_2_, the hydroxyl and phosphate groups being partly substituted by carbonate. INS enables us to detect any changes in the H-bond pattern within this crystalline framework, through access to both the low and high frequency regions of the spectra with good sensitivity.

The bone matrix undergoes macroscopic changes (e.g., in colour and dimensions) upon heating, as well as structural variations, which interfere with the reliability of the available osteometric parameters that are commonly used for the characterization and identification of skeletal remains (e.g., in forensic and archaeological investigations). In the last few years, INS measurements that were carried out at the ISIS Pulsed Neutron and Muon Source of the Rutherford Appleton Laboratory, UK [56] yielded innovative results for several types of human bones [47,48,49,57,58]—femur, humerus, and tibia (osteometrically most informative). These data, combined with those obtained by optical vibrational spectroscopy, delivers the complete vibrational profile of the bone samples, leading to reliable conclusions on the heat-prompted chemical and structural variations as a function of burn temperature—namely regarding the loss of bone´s organic constituents and the microcrystalline changes within the bone matrix. As clearly revealed by these studies, the application of FTIR and Raman techniques is insufficient for accurately detecting all of the vibrational modes of interest in these types of systems and, therefore, retrieve all possible information on their structural preferences and composition, either for intact (unburned) or burned bones.

### 2.4. (n,Υ)-Based Techniques (Radiative-Capture Techniques)

**Figure 4:**
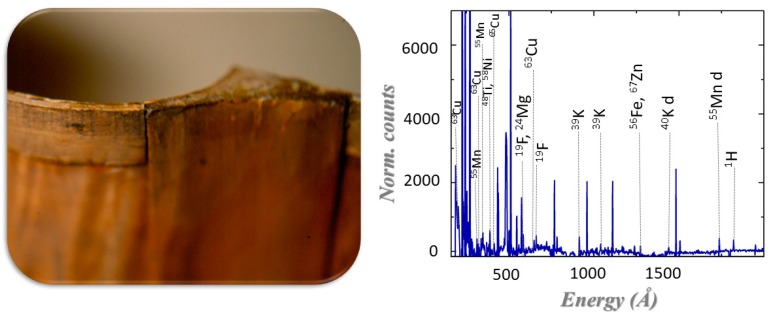
Prompt gamma activation analysis (PGAA) (**Right**) of a wooden sample that has been treated with surface coatings (**Left**).

Radiative-capture techniques, which are also referred to as (n, Υ)-techniques, can be divided into PGAA (prompt gamma activation analysis), NAA (neutron activation analysis), and NRCA (neutron resonance capture analysis). PGAA and NRCA are based on nuclear reactions that take place when a nucleus absorbs a neutron: the compound nucleus is in an excited state and decays to its ground state in 10^−9^−10^−12^ s emitting 2–4 Υ-rays, called prompt gammas. For all of these techniques, which are used for quantitative analysis, the Υ-ray energy is characteristic of the emitting nuclide and their intensities are proportional to the number of nuclei present in the irradiated volume. The key characteristics of the radiative capture of neutrons is that the captured and emitted particles are both highly penetrating and are used for the identification of major, minor, trace, and rare elements. In PGAA, the emitted gamma rays are detected during the neutron irradiation of the sample [59]; an example is shown in Figure 4. PGAA spectra are mostly analyzed through proprietary software such as Hypermet PC [60] and the analysis assumes the validity of the following equation:

(4) $$A_{\gamma }=t\cdot \frac{mN_{A}}{M}\cdot \varepsilon \left(E_{\gamma }\right)\int_{E_{min}}^{E_{max}}\sigma_{\gamma }\left(E\right)\;\varphi \left(E\right)\;f\left(E,\;E_{\gamma }\right)dE$$

where $A_{\gamma }$, is the measured gamma-peak area at energy $E_{\gamma }$, m is the mass of the element that emitted the gamma-ray, *t* is the acquisition time during irradiation, $N_{A}$ is Avogadro’s number, *M* the molar mass, $\sigma_{\gamma }$(*E*) the gamma-ray cross-section and φ(*E*) the flux of neutrons of energy *E*, $\varepsilon \left(E_{\gamma }\right)$ the detector efficiency, and $f\left(E,\;E_{\gamma }\right)$ the neutron and gamma self-absorption correction factor [61]. NAA records the gamma spectrum of the delayed Υ-rays after irradiation in a shielded environment. After the capture of the neutron, the isotope nucleus in the ground state can be either stable or decay with a decay time that is particular of each isotope, and it can vary between minutes (^66^Cu), hours (^64^Cu), days (^198^Au), or years (^60^Co). One can iteratively isolate the elements with a longer decay time by recording several gamma emission spectra of an irradiated sample.

NAA was applied for the first time to Cultural Heritage in 1960, when Emeleus and Simpson published the first results of a study of a Roman pottery fragments to distinguish between production factories [62]. As opposed to PGAA and NAA, where the energy of the decay photons is measured by summing over all of the energies of the captured neutrons, NRCA analyses the peaks in the energy-dependent neutron cross section, summing over all the photon energies emitted in the decay cascade (see Figure 1 for Au). NRCA uses the resonance absorption of neutrons at epithermal energies [63,64] to identify and quantify the isotopes in the irradiated samples, thanks to the time structure of the neutron beams at spallation neutron sources. Resonances are observed by detecting the prompt-gamma radiation that is directly emitted after neutron capture as a function of the neutron energy. In recent years, attempts to measure both the neutron and gamma energies at the same time have been presented [65,66,67,68], in the so-called bi-parametric configuration, and one can foresee an ample range of applicability to Cultural-Heritage studies.

A fascinating example of the use of NAA is provided by the Autoradiography method [69]. In this procedure, a painting is irradiated with neutrons, being possibly slightly tilted with respect to the neutron beam direction, so as to provide a larger surface. Owing to the high-penetration character of neutrons, all of the layers of the painting are irradiated, including those deeper than ca. 150 μm, the analytical depth for X-ray fluorescence. By exposing X-ray films to the irradiated painting, a one-to-one bi-dimensional map of the distribution of the activated elements, related to the pigments used by the artist, can be achieved. By alternating X-ray films at different times after irradiation, one can separate the signals from short-lived isotopes from those with longer decay times [69].

## 3. Neutrons Applied to Cultural Heritage—Case Studies

### 3.1. “The Gates of Paradise” Studied by Neutron Techniques

**Figure 5:**
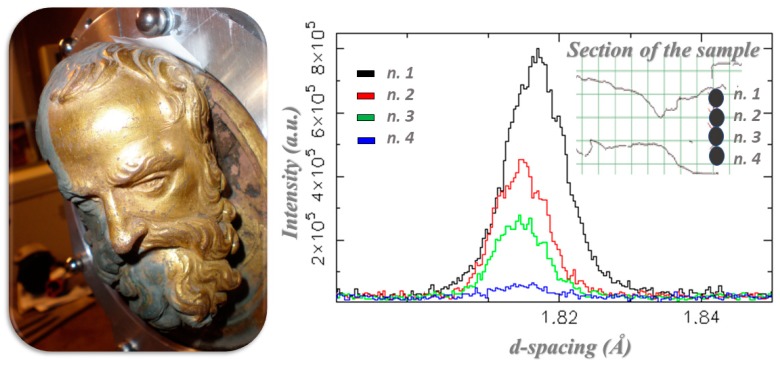
(**Left**) The “prophet head”, by Lorenzo Ghiberti, part of the gilded-bronze door of the Florence baptistery; (**Right**) diffraction patterns of strain scan along the base-plate vertical section of the relief [76].

“The Gates of Paradise” is the name of the gilded east door of the Florence baptistry made by Lorenzo Ghiberti and built between 1425 and 1452. Michelangelo named them the “Gates of Paradise” because of their beauty, and they are known as one of the most famous works of the Florentine Renaissance. They were made of a bronze core with mercury-amalgam gilding on the surface, and were composed of different pieces. Unfortunately, they were damaged during the flood of 1966, resulting in a critical conservation need due to the instability of the gold layer on the bronze alloy. Currently, the doors are kept, and displayed, at the Opera del Duomo Museum in Florence. Several neutron methods were used to study two pieces that depict two prophet heads in order to find out about critical aspects regarding the gilding, to obtain details about the extension of the casting, and understand why such a process was carried out (see Figure 5) [70,71,72,73,74]. Neutron diffraction and neutron radiography were used to obtain valuable information regarding the manufacturing technique and state of conservation of the objects and, in particular, to assess the effectiveness of the two different cleaning techniques, laser cleaning, and chemical bath, being used by the curators during the restoration process [75,76]. The PGAA carried out at Forschungs-Neutronenquelle Heinz Maier-Leibnitz (FRM II) [75] showed that some unstable salts, in particular chlorides, were present in the uncleaned section, but they were also present in the laser cleaning section, while the chemical bath had successfully removed the unwanted compounds. Chlorides were located between the bronze core and the gilding, and they had been responsible for damage to the gilding and pitting of the external surface of the masterpieces. These results were very useful for conservators, which enabled them to plan the microclimatic conditions for a public exhibition of the entire doors.

Neutron diffraction that was carried out on the ENGINX instrument at ISIS [76] shed new light on the nature of the secondary casting of bronze piece representing the prophet’s head: the extension of the hollow volume and the extension of this secondary casting were detected. Additionally, the nature of two rather different castings was identified: the first one implied thermal treatment while the second one was as cast. This evidence suggested that the second casting was made to compensate for a problem that occurred during the first casting process [76].

### 3.2. Egyptian Grave Goods of Kha and Merit Studied by Neutron and Gamma Techniques

**Figure 6:**
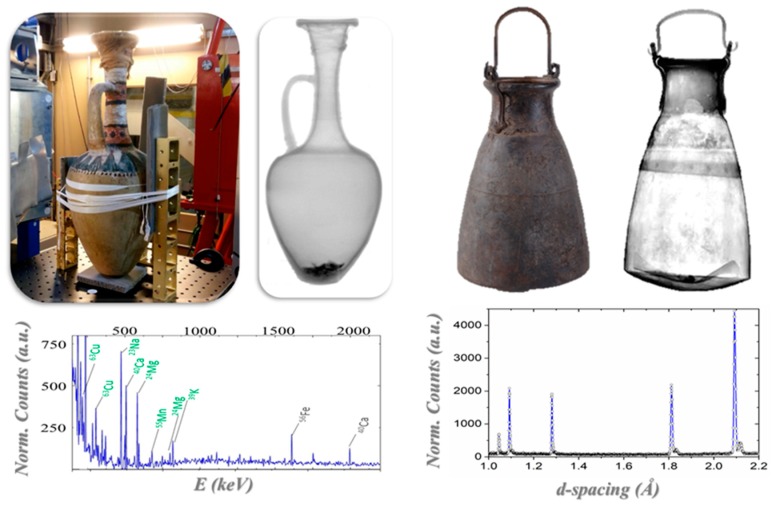
Egyptian objects from the Kha and Merit grave goods. Left: sealed ceramic vase investigated through neutron techniques, neutron radiography, and PGAA plot with the labels of the detected isotopes [32,79]; Right: Egyptian metallic vase (situla), neutron radiography and one of the acquired diffraction patterns [25].

The grave goods of the ancient Egyptian architect Kha and his wife Merit [77,78], preserved at the Museo Egizio in Turin, represent the richest and most complete non-royal burial assemblage housed in a museum outside of Egypt. They date from 1425–1353 BC and they were discovered in 1906 in Egypt in the necropolis of Deir el-Medina, near Luxor. The collection includes alabaster vases, metallic vessels, wooden boxes, tissues, jars for foods, oils, powders and perfumes. Neutron techniques were used, in combination with other physical techniques, such as X-ray based techniques and spectroscopies, to investigate and characterize the collection. A sealed alabaster vase and two sealed pottery vases were analyzed through neutron radiography and tomography, providing a morphological reconstruction of the inner structure of the vases (see Figure 6) [32,79]. Through neutron tomography, the research team was able to carry out a study of the linear coefficients for each component and, thus, distinguish components, such as an alabaster plug under the linen strips that is not visible from the outside and the presence of contents. Neutron tomography revealed that this content is made of an organic compound and the data are compatible with it being a mixture of oils and wax [32]. While using PGAA, elements in the alabaster vase were identified: calcium, sulfur, and oxygen, while carbon, oxygen, and magnesium were identified as part of the organic content of the sealed vase. The investigation of the two large pottery vases showed that they are different in content and in the plug: the first one appeared to be full, with an intact plug under the linen strips; the second one was empty and it did not have an intact plug under the linen strips as well as presenting some fragments in the bottom region that could be interpreted as fragments of the lost plug. A 3D structure of these fragments was also obtained through neutron tomography of the lower region of this pottery vase.

Neutron techniques were also employed to study a metallic vase, an Egyptian *situla*, coming from the same archaeological context: these analyses showed that it was composed of two halves that were held together by rivets. Additionally, the upper part was opaquer to neutrons and this could be attributed to a thicker vase-wall, or to the presence of elements with higher neutron attenuation properties, such as organic residues. PGAA carried out in the upper and lower regions demonstrated that the upper part shows additional peaks, which were compatible with the presence of a bitumen component. Neutron diffraction of the metallic *situla* was carried out at ENGINX at ISIS. The phase analysis and peak broadening analysis showed that the two halves are made of different bronze alloys with different tin contents and are both cold worked [32].

Another study on ancient Egyptian objects, also from the Egyptian Kha and Merit collection [79], was about inks and textiles. The study of Egyptian ink is relevant for archaeologists and historians, for the development of black inks, has enabled writing to become an established method of communication in history. Until recently, little attention had been paid to the nature and technology of inks used on ritual and daily-use textiles, which might have fostered the transfer of metallic ink technology onto other supports, such as papyrus. In this study a combination of X-ray, fluorescence, and Raman spectroscopies, together with PGAA, was applied in order to identify the nature of the black ink. Three different components of the inks were identified: the principal component was iron oxide, a secondary component was manganese oxide, and the base carrier of the colour was probably ochre [80].

### 3.3. A Human Skeleton from the Roman Period Probed by Neutron Spectroscopy

Bones that have been subjected to heating are commonly found in archaeological sites, mainly as a result of ancient burial practices. An innovative approach for the study of this type of human skeletal remains is based on neutron techniques, and it has provided archaeologists and bioanthropologists with useful information regarding past civilizations (namely regarding funerary and cooking habits) as well as on the environmental setting of the samples [47,81].

**Figure 7:**
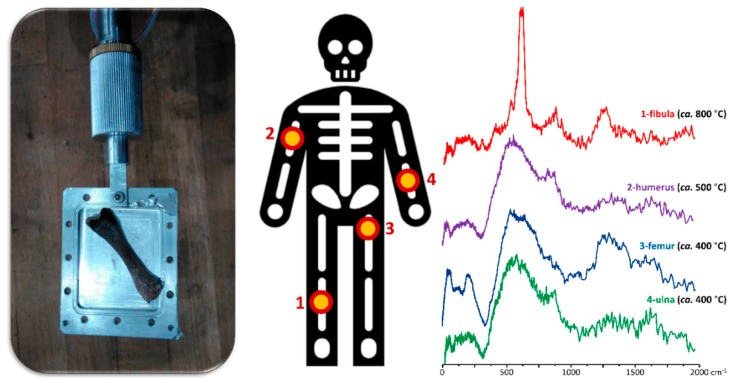
(**Left**) human bone in the sample holder; middle: location in the skeleton of the bones studied; and (**Right**) vibrational spectra of samples coming from this same skeleton (Leopoli-Cencelle, Italy), over a wide range of firing temperatures (400 °C to 800 °C) [57,82].

In particular, regarding an INS study on a human skeleton from the Roman period, which was found at the archaeological Guidonia site (unwrapped inside an earth tomb), it was possible to infer the specific heating conditions to which these ancient bones were subjected based on results previously gathered for modern bones burned under controlled conditions (Figure 7) [47]. This allowed the archaeologists to identify funerary and burial practices as well as to better characterize the archaeological site where the bones were found (namely the chemical composition of the soil).

To date, this kind of neutron spectroscopic fingerprints of ancient skeletal remains have been obtained for different archaeological contexts spanning the Neolithic to the Roman Period and the Middle Ages [47,82].

## 4. Conclusions

The successful application of neutron techniques in Cultural Heritage is demonstrated by the growing impact of neutron techniques applied to archaeology, as well as in the restoration and conservation of historical artifacts (e.g., paintings, sculptures, and ceramics). A synergetic combination of neutron techniques enables us to unveil new information regarding ancient objects. Neutron-based experimental methods are expected to generate an impact across several disciplines in the field of Cultural Heritage, such as archaeology, anthropology, and conservation/restoration.

The unique properties of neutrons provide results and insights that are not possible to attain with other experimental methods and open new opportunities for studying past civilizations. An active collaboration between researchers and experts from different disciplines, such as archaeology, museology, and scientific research, through a constructive dialogue, will be invaluable to shed light into many unsolved questions about our Heritage via the intersection of different skills and methodologies. Today, neutron techniques are already being used on a regular basis for enriching the study of samples from public museums and private collections. The results of these collaborative efforts are being increasingly presented to the general public at museums as an added value to the exhibitions, which makes visitors aware of the discoveries made on particular objects of the collections by these newly available physical-chemical tools.
